# Supplementary material for: Structural basis of nucleic-acid recognition and double-strand unwinding by the essential neuronal protein Pur-alpha
Source: eLife. 2016 Jan 8;5:e11297. doi: 10.7554/eLife.11297 (PMC4764581; doi:10.7554/eLife.11297)
Supplement: Figure 6—source data 1. — Amino acids in brackets indicate the corresponding positions in Drosophila Pur-alpha, based on the protein-sequence alignment shown in Figure 2—figure supplement 4. “p.” indicates a point mutation, “*” a stop codon, “fs” a frame shift followed by the number of residues produced until the next stop codon, “del” a deletion, and “delins” a combination of deletion and insertion. DOI: http://dx.doi.org/10.7554/eLife.11297.020 [file elife-11297-fig6-data1.docx]

| **Reference** | **Mutation** | **Predicted structural effect** |
| --- | --- | --- |
| Lalani et al. 2014 | p.F271del  (*D.m*.: F246) | Deletes C-terminus. |
|  | p.S103H fs*97  (*D.m*.: A86) | Deletes part of PUR repeat I, the entire PUR repeats II and III, and C-terminus. |
|  | p.Q186*  (*D.m*.: S159) | Deletes part of PUR repeat II, the entire PUR repeat III and C-terminus. |
|  | p.L97E  (*D.m*.: R80) | Affects PUR repeat I: Likely impairs DNA/RNA binding (**Figure 6B**). |
|  | p.L100P  (*D.m.*: I83) | Affects PUR repeat I: Likely impairs beta-sheet formation and hydrophobic core. |
|  | p.Y121*  (*D.m.*: Y104) | Deletes part of PUR repeat I, the entire PUR repeats II and III, and C-terminus. |
|  | p.Y261*  (*D.m.*: E236) | Deletes part of PUR repeat III and entire C-terminus. |
|  | p.M157L  (*D.m.*: L135) | Affects PUR repeat II, effect unclear. |
|  | p.A89P  (*D.m.*: A72) | Affects PUR repeat I: Likely impairs DNA/RNA binding (**Figure 6A**). |
|  | p.I88_A89 delins T  (*D.m.*: V71_A72) | Affects PUR repeat I: Likely impairs DNA/RNA binding. |
|  | p.R199P  (*D.m.*: R172) | Affects PUR repeat II: Likely impairs DNA/RNA binding and folding of beta-sheet of the PUR repeat. |
| Hunt et al. 2014 | p.F243Y fs*50  (*D.m.*: Y218) | Destroys PUR repeat III and C-terminus. |
|  | p.E283R fs*45  (*D.m.*: D258) | Destroys C-terminus. |
|  | p.I206F  (*D.m.*: L179) | PUR repeat II: Likely impairs intramolecular dimerization of repeats I and II. |
| Hunt et al. 2014 &  Tanaka et al. 2015 | p.F233del  (*D.m.*: F208) | PUR repeat III: Likely impairs dimerization of Pur-alpha. |
| Tanaka et al. 2015 | p.I188T  (*D.m.*: I161) | Affects PUR repeat II: Likely impairs dimerization with PUR repeat I. |
|  | p.I257H fs*37  (*D.m.*: I232) | Destroys PUR repeat III and C-terminus. |
|  | p.M1 no_start | Probably no protein produced because of mutation of start codon. |
|  | p.A2P fs*197 | Alters the sequence of almost the entire protein. |
|  | p.T101_S103del  (*D.m.*: Y84_A86) | Affects PUR repeat I: Likely impairs dimerization with repeat II. |

**Figure 6-source data 1.** Mutations in the gene encoding for human Pur-alpha that result in the 5q31.3 microdeletion syndrome. Amino acids in brackets indicate the corresponding positions in *Drosophila* Pur-alpha, based on the protein-sequence alignment shown in Figure 2-figure supplement 4. “p.” indicates a point mutation, “*” a stop codon, “fs” a frameshift followed by the number of residues produced until the next stop codon, “del” a deletion, and “delins” a combination of deletion and insertion.
